# Genomic nucleotide-based distance analysis for delimiting old world monkey derived herpes simplex virus species

**DOI:** 10.1186/s12864-020-06847-w

**Published:** 2020-06-26

**Authors:** Aaron W. Kolb, Curtis R. Brandt

**Affiliations:** 1 Department of Ophthalmology and Visual Sciences, School of Medicine and Public Health, University of Wisconsin-Madison, 550 Bardeen Laboratories, 1300 University Ave, Madison, WI 53706 USA; 2 McPherson Eye Research Institute, University of Wisconsin-Madison, Madison, WI USA; 3 Department of Medical Microbiology and Immunology, School of Medicine and Public Health, University of Wisconsin-Madison, Madison, WI USA

**Keywords:** Virus, Herpes, Species, Cryptic species, Genome, Phylogeny, Species delimiting, Macaque

## Abstract

**Background:**

Herpes simplex viruses form a genus within the alphaherpesvirus subfamily, with three identified viral species isolated from Old World monkeys (OWM); *Macacine alphaherpesvirus 1* (McHV-1; herpes B), *Cercopithecine alphaherpesvirus 2* (SA8), and *Papiine alphaherpesvirus 2* (PaHV-2; herpes papio). Herpes B is endemic to macaques, while PaHV-2 and SA8 appear endemic to baboons. All three viruses are genetically and antigenically similar, with SA8 and PaHV-2 thought to be avirulent in humans, while herpes B is a biosafety level 4 pathogen. Recently, next-generation sequencing (NGS) has resulted in an increased number of published OWM herpes simplex genomes, allowing an encompassing phylogenetic analysis.

**Results:**

In this study, phylogenetic networks, in conjunction with a genome-based genetic distance cutoff method were used to examine 27 OWM monkey herpes simplex isolates. Genome-based genetic distances were calculated, resulting in distances between lion and pig-tailed simplex viruses themselves, and versus herpes B core strains that were higher than those between PaHV-2 and SA8 (approximately 14 and 10% respectively). The species distance cutoff was determined to be 8.94%, with the method recovering separate species status for PaHV-2 and SA8 and showed that lion and pig-tailed simplex viruses (vs core herpes B strains) were well over the distance species cutoff.

**Conclusions:**

We propose designating lion and pig-tailed simplex viruses as separate, individual viral species, and that this may be the first identification of viral cryptic species.

## Background

The alphaherpesvirinae comprise a subfamily within Herpesviridae, with most of its members establishing latency in the peripheral nervous system. The five genera which comprise the alphaherpesvirinae infect birds (*Iltovirus*, *Mardivirus*), sea turtles (*Scutavirus*), mammals (*Varicellovirus*, *Simplevirus*), as well as lizards (currently unassigned). Until fairly recently, simplex viruses were thought to only infect primates, however simplex viruses have been isolated from cattle, bats, rabbits, and marsupials [1–5]. Various species of macaque monkeys are the natural reservoir for the herpes B simplex virus. Herpes B was first described in 1933, following an incident where a 29-year-old laboratory worker was bitten by an asymptomatic monkey and later died from encephalitis [6, 7]. Herpes B has been demonstrated to be highly neurovirulent with ~ 80% mortality and is categorized as a BSL-4 level pathogen by the CDC [8, 9]. In spite of considerable work with macaques in laboratory settings, as well as close contact between humans and macaques particularly in Asia, there have only been 46 documented cases of zoonotic transmission since 1933 [10, 11]. A recent commentary has questioned the high neurovirulence of herpes B and has raised the possibility of higher rates of viral shedding in laboratory settings due to stress [11].

Herpes B has an approximately 156,400 bp genome, a high GC content of 74.5%, and has been shown to be closely related to *Papiine alphaherpesvirus 2* (PaHV-2; herpes papio) and *Cercopithecine alphaherpesvirus 2* (SA8). With the advent of next-generation sequencing (NGS) the genomes of 19 herpes B isolates have been sequenced [12–14]. The sequenced strains were isolated from six macaque species; *Macaca* (*M.) fascularis* (crab-eating; cynomologous; cyno), *M. fuscata* (Japanese), *M. mulatta* (rhesus)*, M. nemestrina* (pig-tailed), *M. radiata* (bonnet), and *M. silenus* (lion-tailed). Macaque phylogenetic research has shown that of the macaque species featured in the current study, *M. silenus* and *M. nemestrina* are basal to the remaining species [15]. A herpes B multi-isolate analysis previously showed that herpes B strains isolated from *M. silenus* and *M. nemestrina* were distant from the remaining macaque derived sequences according to percent coding identity [12].

For several decades, the classic definition of species originating from Ernst Mayr has been “species are groups of actually or potentially interbreeding natural populations, which are reproductively isolated from other such groups” [16, 17]. This definition is problematic in virology as viruses undergo recombination [18–26], but they do not interbreed per se, so an alternative definition is required. The definition of species has not been static, with several alternative species concepts proposed based on biological, ecological, evolutionary, cohesion, phylogenetic, phenetic, and genotypic cluster properties, many of which have further subdivisions [27]. Related to challenges regarding species concepts, are cryptic species (non-viral) which have been described since the early eighteenth century [28, 29]. Cryptic species appear identical based on morphology but are on different evolutionary paths [29]. The definition of cryptic species lacks clarity, however, a recently proposed conceptual framework for identifying cryptic species involves “statistically separable and divergent genotypic clusters” [29]. To address these challenges several methods of species delimitation have been used in organisms ranging from bacteria to eukaryotes such as arbitrary distance thresholds, in silico DNA-DNA hybridization (isDDH) and generalized mixed Yule coalescent (GMYC) [30–33]. Previous phylogenetic studies of porcine circovirus type 2 (PCV2), H5N1 influenza, *feline herpesvirus 1* (FHV-1), and the varicellovirus genus have used genomic nucleotide distance to establish intraspecies clade cutoffs [34–37]. The goal of the current study was to use this genomic distance cutoff approach to determine if the herpes B strains isolated from *M. silenus* and *M. nemestrina* constituted cryptic viral species, warranting species status.

## Results

### Old world monkey simplex virus phylogeny

**Fig. 1:**
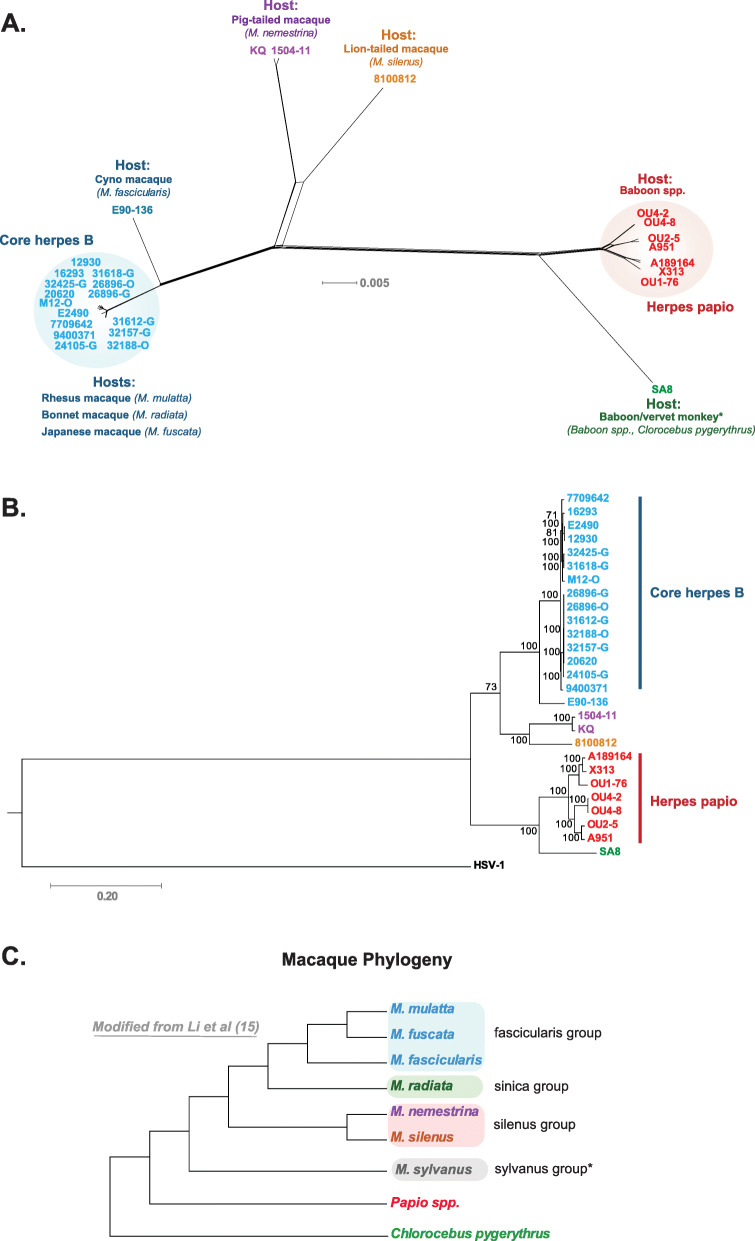
Phylogenetic analysis of Old World monkey (OWM) derived simplex viruses. OWM viral genomic sequences (Table 1) were aligned with MAFFT ver. 7.394 and the optimal substitution model was calculated by IQ-Tree [42, 43]. **a** Phylogenetic network generated from the alignment using Splitstree ver. 4.14 and the HKY + G + I substitution model (gaps deleted; p-inv = 0.469; gamma = 1.138) [44] was used. **b** Maximum Likelihood tree was generated from an alignment using HSV-1 as an outgroup using RAxMLGUI (GTRCATI; ver 1.3) [45]. Figure **c** shows a macaque monkey phylogenetic tree based on data presented by Li et al. [15]

To investigate if the pig and lion-tailed macaque simplex viruses warranted separate species status, the genomes of the available Old World monkey (OWM) derived simplex viruses were downloaded from Genbank (Table 1). The available PaHV-2 strains were included in the analysis in order set an overall species cutoff for the OWM simplex viruses. The viral genomes were first aligned, and then the terminal repeat segments were deleted from the genomic multiple sequence alignment (MSA). The optimal nucleotide substitution model for the dataset was also calculated. This MSA alignment was used to generate a phylogenetic network which illustrates phylogenetic dissonance within the dataset (Fig. 1a). The phylogenetic network in Fig. 1a shows a “genetic continuum” with the core herpes B strains at one end, the pig and lion-tailed macaque derived strains located approximately in the middle, and the baboon viruses at the opposite end of the continuum. Additionally, the herpes B strain E90–136, isolated from a cyno macaque was separated from the core herpes B strains. A maximum likelihood (ML) tree was also generated to establish phylogenetic robustness, and the subsequent tree produced highly similar results to phylogenetic network (Fig. 1b). The OWM simplex virus phylogenetic network and ML tree (Fig. 1a and b) show similar phylogenetic tree topology to the Old World monkey hosts (Fig. 1c).

**Table 1:** The abbreviations, synonyms, strains, hosts, genome lengths, and accesion numbers for the viruses used in the current study

Abbreviation	Synonym	Strain	Host	Genome length	Accession number
HSV-1	Herpes simplex virus type 1	17	*Homo sapiens*		NC_001806.2
CeHV-2	SA8	B264	*Cercopithecus aethiops*^a^	150,715	NC_006560.1
HVP-2	Herpes papio	X313	*Papio cynocephalus*	156,487	NC_007653.1
HVP-2	Herpes papio	OU4–2	*Papio ursinus*	138,963	KF908244.1
HVP-2	Herpes papio	OU4–8	*Papio ursinus*	139,193	KF908243.1
HVP-2	Herpes papio	A951	*na*	138,559	KF908242.1
HVP-2	Herpes papio	OU2–5	*Papio cynocephalus*	138,807	KF908241.1
HVP-2	Herpes papio	OU1–76	*Papio cynocephalus*	148,944	KF908240.1
HVP-2	Herpes papio	A189164	*na*	139,366	KF908239.1
CeHV-1	Herpes B	E2490	*Macaca mulatta*	156,789	NC_004812.1
CeHV-1	Herpes B	M12-O	*Macaca radiata*	155,404	KY628985.1
CeHV-1	Herpes B	9400371	*Macaca mulatta or* *fascicularis*^b^	155,143	KY628983.1
CeHV-1	Herpes B	7709642	*Macaca fuscata*	155,141	KY628982.1
CeHV-1	Herpes B	32425-G	*Macaca mulatta*	155,528	KY628981.1
CeHV-1	Herpes B	32188-O	*Macaca mulatta*	155,099	KY628980.1
CeHV-1	Herpes B	32157-G	*Macaca mulatta*	155,777	KY628979.1
CeHV-1	Herpes B	31618-G	*Macaca mulatta*	155,425	KY628978.1
CeHV-1	Herpes B	31612-G	*Macaca mulatta*	155,321	KY628977.1
CeHV-1	Herpes B	26896-O	*Macaca mulatta*	155,583	KY628976.1
CeHV-1	Herpes B	26896-G	*Macaca mulatta*	155,609	KY628975.1
CeHV-1	Herpes B	24105-G	*Macaca mulatta*	155,021	KY628974.1
CeHV-1	Herpes B	20620	*Macaca mulatta*	155,323	KY628973.1
CeHV-1	Herpes B	16293	*Macaca mulatta*	155,180	KY628972.1
CeHV-1	Herpes B	12930	*Macaca mulatta*	155,462	KY628971.1
CeHV-1	Herpes B	KQ	*Macaca nemestrina*	157,321	KY628970.1
CeHV-1	Herpes B	1504–11	*Macaca nemestrina*	156,905	KY628969.1
CeHV-1	Herpes B	8100812	*Macaca silenus*^c^	157,447	KY628968.1
CeHV-1	Herpes B	E90–136	*Macaca fascicularis*	155,157	KJ566591.2

^a^Subsequent studies following isolation show that the natural reservoir for SA8 is baboons [38–40]

^b^Host species differs between the Genbank annotation and the corresponding publication [12]

^c^Strain was originally isolated from *C. neglectus*, however subsequent work showed the natural reservoir is *M. silenus* [41]

### Establishing species level cutoffs

**Fig. 2:**
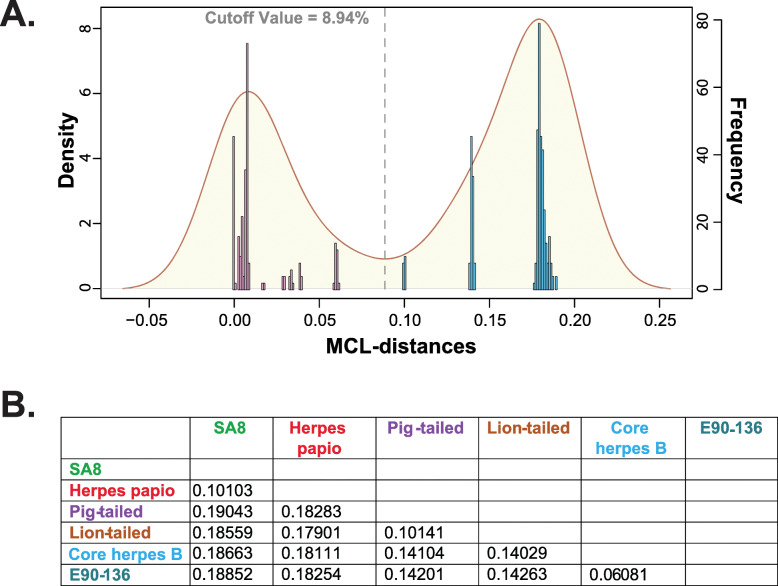
Establishing viral species cutoff value. Pairwise distances in the Old World monkey virus alignment were calculated using Mega 7 [46], and the frequencies plotted using the R package. A kernel density plot was also generated and combined with the distance frequencies (**a**). A distance cutoff value was established by determining the trough of the kernel plot, which is depicted by a vertical dotted line (8.94%). Mega 7 was used to calculate between group distances which is shown in Figure **b**

Genomic nucleotide distance-based cutoff values have been used in the past in an effort to define viral intraspecies clades empirically [34–37]. In the current study we applied this distance-based method to define species level cutoffs. To begin to establish species level cutoffs, the maximum composite likelihood (MCL) pairwise distances between the 28 OWM viruses was calculated, the frequencies plotted, and a kernel density graph was overlaid (Fig. 2a). A genomic distance cutoff for establishing species status was derived by marking the lowest point of the kernel density plot (8.94%) and is denoted by the vertical dashed line in Fig. 2a. Thus, for the current data set, genomic distances over 8.94% merit species status, and under 8.94% do not. Using this genomic nucleotide-based distance cutoff approach, the pig and lion-tailed macaque simplex viruses merit separate, individual species status, as the distances between each other was 10.1%. The distance of the pig and lion-tailed macaques from the core herpes B strains was approximately 14% (Fig. 2b), suggesting they are separate species. Using this method, SA8 and PaHV-2 retained species status, however the outlying core herpes B isolate E90–136 did not merit species status (6.1% distance; Fig. 2b).

### Core herpes B clade

**Fig. 3:**
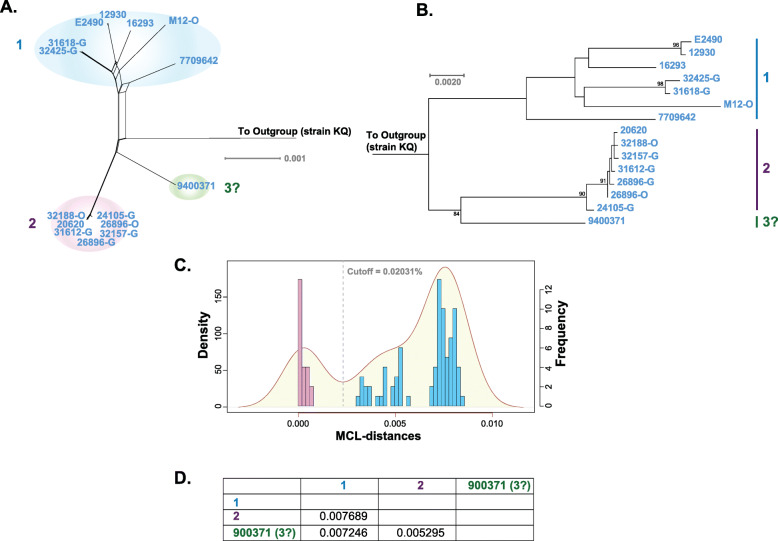
Core herpes B phylogeny and clades. A genome sequence alignment was generated with the core herpes B strains identified in Fig. 1. A phylogenetic network using the HKY + G + I substitution model (gaps deleted; p-inv = 0.686; gamma = 0.927) (**a**) and maximum likelihood tree (**b**) were then produced, finding three provisional clades. Pairwise distances between the strains were plotted (shown in Figure **c**) and a clade cutoff value (vertical dotted line) was calculated (0.0203%). Figure **d** contains a table showing the between group genetic distances

The core herpes B strains isolated from rhesus, bonnet, and Japanese macaques were next examined to establish intraspecies genomic distance-based clade cutoff. Similar to the method described above, MSAs comprising the 15 core herpes B strains identified in Fig. 1a and b were generated with and without an outgroup (*M. nemestrina* isolate KQ). Next, a phylogenetic network and maximum likelihood tree were constructed (Fig. 3a and b) based on the alignment with an outgroup. The tree topology patterns between the two phylogenetic methods were nearly identical, with two basic groupings, aside from an outlier strain (9400371). Next, pairwise distances between the core herpes B strains were calculated using the core herpes B MSA without an outgroup, and the frequencies were plotted (Fig. 3c). The genomic distance clade cutoff derived from the kernel density trough was 0.2031% (Fig. 3c). The distance between groups 1 and 2 was 0.7689% (Fig. 3d), which is above the distance cutoff validating their status as clades. The distance between strain 9400371 and clades 1 and 2 was 0.07246 and 0.05295% respectively, therefore because these values are above the 0.02031% cutoff value, strain 9400371 may warrant consideration as a single member of a third clade.

### PaHV-2 clade structure

**Fig. 4:**
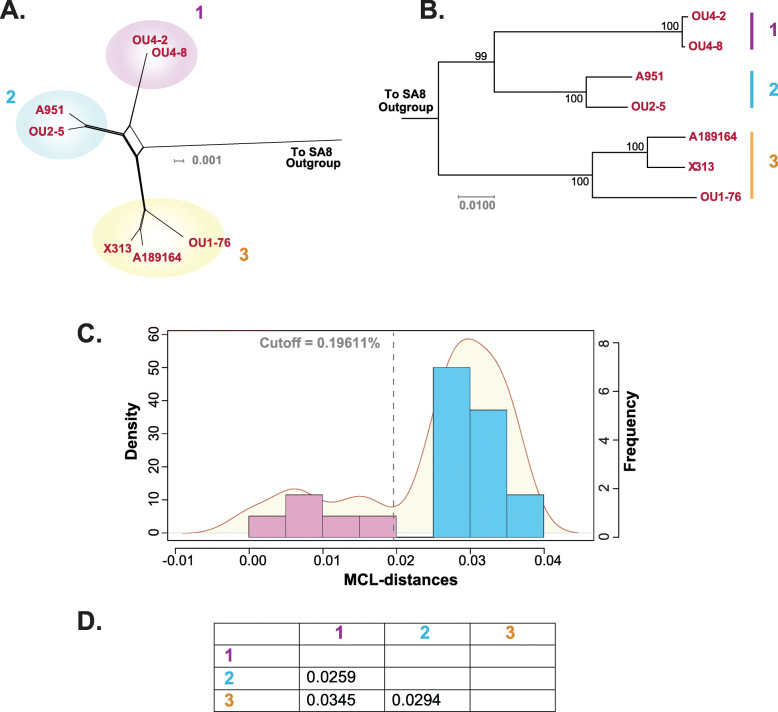
PaHV-2 phylogeny and clades. A genome sequence alignment was generated with the available PaHV-2 strains (Table 1). A phylogenetic network (Figure **a**) was generated using the HKY + G + I substitution model recommended by IQ-Tree (gaps deleted; p-inv = 0.572; gamma = 0.739). Figure **b** shows a maximum likelihood tree which shows three clades. Pairwise distances between the strains were plotted (Figure **c**) and a clade cutoff value calculated (1.96%). Figure **d** includes a table showing the between group genetic distances

The phylogenetic structure of the seven available PaHV-2 genomic sequences was examined examined. Both the phylogenetic network and maximum likelihood tree recovered three groupings (Fig. 4a and b). The clade cutoffs were performed in the same manner as described above, with the cutoff value calculated at 1.9611% distance (Fig. 4c). The distances between groups 1, 2 and 3 were above the cutoff (Fig. 4d), thus validating their clade status.

## Discussion

In the current study we utilized a genomic nucleotide distance-based method previously used for identifying phylogenetic clades and applied it to detect viral species. The results suggest that herpes simplex viruses isolated from lion and pig-tailed macaques should be designated as separate species. To our knowledge this is the first time this technique was been applied to virus species and may be useful in detecting cryptic viral species.

### Host-virus co-speciation

**Fig. 5:**
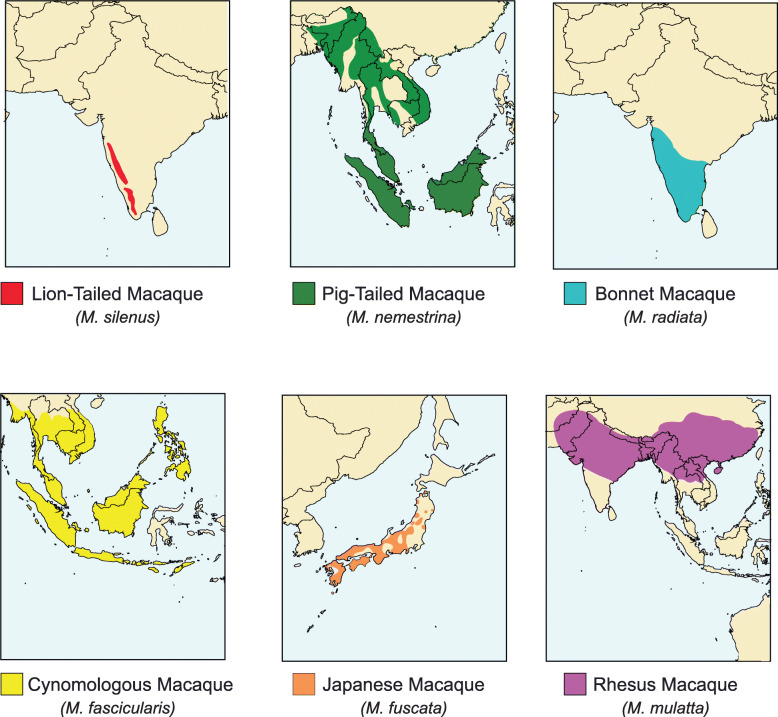
Maps depicting macaque species ranges. The figure shows the natural ranges for the pig-tailed, lion-tailed, bonnet, crab eating/cynomologous, Japanese and rhesus macaques. The maps were generated in R (version 3.4.2, “maps” package), with colors and species ranges added using Adobe Illustrator. The maps were based on those available from Wikimedia Commons, and are free use upon citing the authors of the images in accordance with the Creative Commons license (https://creativecommons.org/licenses/by-sa/3.0/deed.en). The authors of all the Wikimedia Commons maps were Chermundy and the IUCN Red List (https://www.iucnredlist.org/)

Herpesviruses have been shown to cospeciate with their hosts [47], however they can cross species barriers [48], especially in captivity [38, 39, 41, 49–53]. These captive transmissions, especially between macaque species can complicate phylogenetic analysis. In particular, cross-species transmission appears to be fairly common among the core herpes B strains, and has been discussed previously in depth by Eberle et al. [12]. In some of the herpes B strains, the original source of the virus appears to be unclear. For instance, the cynomolgus macaque derived strain E90–136 is more distant and phylogenetically separated from the core herpes B strains (Fig. 1), however it was not sufficiently distant (Fig. 2) to be considered a separate species. Interestingly, strain E90–136 was isolated from a cyno macaque which died due to a disseminated infection caused by the virus [54]. Herpes B strains are generally asymptomatic within the natural host, which may suggest that cyno macques are not the natural reservoir for this particular viral strain. For other OWM strains, interspecies spread is well documented. The isolate 8100812 was originally isolated from a DeBrazza monkey, however restriction digest patterns showed that the lion-tailed macaque was the natural host [41]. Phylogenetically, this appears appropriate as strain 8100812 forms a node with the two pig-tailed macaque isolates (Fig. 1a and b), and importantly matches phylogenetic profile of the macaque species themselves (Fig. 1c). The correlation between lion and pig-tailed viruses and macaque phylogeny strongly suggests host-virus co-speciation. Additionally, while natural cross-species viral transmissions between animals does occur [48, 55–57], natural species viral transmissions between the animals and viruses in this study are fairly unlikely given the natural host ranges of the monkeys (Fig. 5). The reduced likelihood of natural cross species transmission is important as it increases the probability of host-virus co-evolution. Further, for example while lion-tailed and bonnet macaques ranges overlap, different living strategies (frugivorous and arboreal vs generalist in human dominated environments respectively) [58, 59] between these animals make cross transmission unlikely.

### Viral species concept

Standard definitions of what constitutes a biological species, such as a reproductively isolated population [16], are insufficient for viruses as they replicate, but do not reproduce like other organisms. Originally, viruses were simply classified according to the host that was infected, i.e. bacterial, plant or animal [60]. It wasn’t until 1950 that official principles of animal virus classification were established, with categories such as morphology, chemical composition, method of transmission, tropism and symptomatology [60]. In 1963 the International Committee on Nomenclature of Viruses (ICNV) was established and in 1966 the body proposed a taxonomic framework and classification rules which included class, order, family. This organization is now known as the International Committee for Taxonomy of Viruses (ICTV) [60, 61]. In 1990 the ICTV established an official definition of viral species which was stated as “a virus species is a polythetic class of viruses that constitutes a replicating lineage and occupies a particular ecological niche” [62], and has since evolved to state “a monophyletic group of viruses whose properties can be distinguished from those of other species by multiple criteria … .not limited to natural and experimental host range, cell and tissue tropism, pathogenicity, vector specificity, antigenicity, and the degree of relatedness of their genomes or genes [63]. While this statement recommends distinguishing properties for determining species, the process is still ambiguous.

We chose to focus our efforts on genomic distance in order to apply a quantitative measure to delimit viral species. Several species delimitation methods have been used in bacteria and eukaryotes. One of the most common and recent methods for species delimitation in bacteria and eukaryotes is generalized mixed Yule coalescent, where branching patterns of a single tree transition from Yule process inter-species branching to coalescent process intra-species branching [33]. Single loci can be used for this method, however more recently multiple genes and morphological characters can be used [64]. Previously, a distance method based on gene homology and sharing was used to reevaluate viral family classifications [65]. A relatively simple genomic distance cutoff method has been used to validate viral clades [34–37] and was applied to delimit species in the current study. While the kernel density plot combined with genetic distance cutoff method described here is simplistic compared to the computation heavy generalized mixed Yule coalescent method, whole genomes and therefore more phylogenetic signal is available for analysis. We did not compare the various species delimiting methods to the genetic distance cutoff method as this was beyond the focus of the study. A caveat with the distance cutoff value used in the current study is that the cutoff value is not universal, but dataset dependent. A potential issue with using the distance cutoff method to establish species boundaries is that as the genomes of additional viruses are sequenced, the species cutoff value could potentially shift, resulting in species cutoff values that could vary over time. A general complication of the method used in the current study and in other genetic data delimitation techniques is that the methods may be delimiting populations, and not necessarily species [66]. We cannot eliminate this possibility in our analysis however this is unlikely given the large distance values between species in the dataset.

In our study to determine if the lion and pig-tailed derived simplex viruses were species separate from herpes B, we included all sequenced Old World monkey strains in an effort not to bias the results and establish a general cutoff for the Old World monkey group. The results of our study showed the genome-based genetic distance between lion/pig-tailed macaque derived viruses and the core herpes B strains were both approximately 14%, which was actually greater than the distance observed (~ 10%) between SA8 and PaHV-2 (Fig. 2b), previously established viral species. The recovery of SA8 and PaHV-2 as separate species helps to validate the method. Both of these values were well above the species cutoff value (8.94%; Fig. 2b). The genetic distance data, and the data supporting co-speciation of the lion and pig-tailed macaque viruses reinforces the idea that these should be designated as separate, individual species from herpes B, and each other.

### Cryptic viral species

The term cryptic species is related to similar concepts such as sibling species, species complex, and superspecies, with the definitions between these concepts often blurred. Cryptic species are generally defined as species which appear virtually identical phenotypically, but belong to different taxa, and were thus “hidden”. It should be noted that it is not unusual for non-viral cryptic species to have some morphological differences in terms of color, size, and markings [67, 68]. Cryptic species were originally described three centuries ago [28, 29], and with modern molecular techniques have been increasingly identified across multiple organisms [69–73]. To our knowledge, the concept of cryptic species has not been applied to viruses, however species complex occasionally has [74, 75]. From the phylogenetic network of the Old World monkey simplex viruses (Fig. 1a), these viruses could be described as a series of species complexes (i.e. a group closely related viruses that are difficult to separate), one comprising the macaque viruses and a second encompassing the baboon simplex viruses. The genetic distance cutoff method may be useful in establishing species boundaries in these complexes, as the method confirmed species status for the baboon derived PaHV-2 and SA8. Importantly, the method identified lion and pig-tailed simplex viruses as separate species (Fig. 2), defining these viruses essentially cryptic species. The genetic distance cutoff method provides a quantitative threshold to determine species status and could be another tool for establishing species status among viral cryptic species complexes.

### Challenges and issues

There are multiple challenges in defining species, for example recently, even in defined species, fertile hybrids among plants, birds, fish, and even mammals are not uncommon [76–79], suggesting reproductive barriers may not always separate species. This may call into question as to what constitutes a species. As previously stated, viruses do not reproduce per se, however they do recombine, and herpesviruses have been shown to be highly recombinogenic [26, 80]. Several recent studies have found natural interspecies recombinants between HSV-1 and HSV-2 [18, 20], although they share approximately 70% sequence similarity [81]. While natural recombinants between OWM viruses, which have lower genetic distances than HSV-1 and 2 have not been reported, it seems reasonable to assume it is possible. It is therefore unlikely that the ability to recombine in a host would be a factor in defining species in primate herpes simplex viruses.

Species defining methods related to virion morphology, serology, as well as gene homology and function are problematic in primate herpes simplex viruses as these characteristics are highly similar, with one of the only differences being the apparent lack of γ_1_34.5 in the Old World monkey simplex viruses [40]. Virus morphology in particular is difficult to distinguish between simplex viruses, as an older study found that virion morphology is nearly the same between HSV and herpes B, however there may some minor differences in morphogenesis [82]. Further, to our knowledge, differences in virus morphology between in the various herpes B strains has not been investigated. From the studies performed so far, the herpes B strains examined here appear to be nearly identical in nearly every respect, including the ability to infect multiple monkey species. Future studies may be able to detect morphological difference in the viral virions or at the protein structure level. Pathogenicity is one determinative method in which there appears to be a difference between the pig and lion-tailed macaque viruses and the remaining herpes B strains. Studies performed by Eberle et al. examining the lethal dose (LD_50_) of the sequenced herpes B strains in mice showed that the pig and pion-tailed macaque simplex viruses had different lethality phenotypes compared to the remaining herpes B strains [12]. Importantly, the LD_50_ values for the pig and lion-tailed viruses were > 10^7^ PFU, while the average for the remaining herpes B strains was approximately 10^4^ PFU. In addition to the species delimiting method described here, pathogenicity differences support separate species designations for the pig and lion-tailed macaque simplex viruses.

### Implications of separate species designation

There are several related scientific threads derived from giving species designations to the pig and lion-tailed macaque species. The first is acknowledging that these viruses are on separate evolutionary paths from each other, and from other herpes B strains. This may result in closer examination of possible phenotypic differences between herpes B strains, and among other groups of closely related viruses. Further, possible future transcriptomic or proteomic data conclusions from core herpes B strains for example will not be assumed for the pig and lion-tailed herpes simplex viruses and would require separate experimentation.

### Herpes B core phylogeny

Phylogenetic analysis of the remaining herpes B strains showed a core group, designated core herpes B, containing two main clades (Fig. 3a and b). Core herpes B clade 1 contained strains with longer branch lengths compared to clade 2, with strains derived from *M. mulatta*, *M. radiata* (strain M12-O), and *M. fuscata* (strain 7709642). It is unclear why the branch lengths are longer in clade 1, however the isolation locations and host species are variable [12] and may contribute to the greater genetic distances. The strains comprising clade 2 were all isolated from *M. mulatta*, and from two locations. It is possible that clade two represents a rhesus only strain grouping. Herpes B core strain 9400371 may represent a sole member of a third clade, with genetic distances from clades one and two that were above the cutoff threshold (Fig. 3d). The original host for this virus is unclear as the Genbank annotation (KY628983.1) states that it is rhesus macaque, however the corresponding publication [12] states it is from a cynomolgus macaque. If strain 9400371, is derived from a cynomolgus macaque, future research will help determine if is the first member of a cynomolgus macaque clade.

## Conclusion

In conclusion, genome-based phylogenetic and genetic distance cutoff techniques were applied to the available Old World monkey simplex virus genome sequences. The results showed that lion and pig-tailed macaque simplex viruses were approximately 14% distant from core herpes B strains, which was more distant than between PaHV-2 strains and SA8, previously established viral species. The genomic distance cutoff method recovered PaHV-2 and SA8 as separate species, and lion and pig-tailed macaque simplex viruses as separate species, effectively identifying these macaque viruses as cryptic species. Based on the genetic distance analysis, the fact that the OWM hosts are designated as separate species, and herpes viruses co-evolve with their hosts, we propose establishing lion and pig-tailed macaque simplex viruses as separate species. This may be the first identification of cryptic viral species.

## Methods

### Genome sequences and genomic sequence alignment

The genomic sequences of the viral strains used in the current study were downloaded from NCBI and can be found in Table 1. Several genomic multiple sequence alignments (MSAs) were generated with MAFFT (Linux ver. 7.394) using the FFT-NS-1 strategy option [42, 83]. MSAs with and without an outgroup were generated for herpes B, PaHV-2, and all available Old World monkey (OWM) genomic sequences. The generated MSAs were manually inspected, and locally aligned for optimization using ClustalW within the MEGA 7 package [46, 84]. The alignments generated for this study can be downloaded at https://brandt.ophth.wisc.edu/data-sets/.

### Nucleotide substitution model optimization and phylogeny

Prior to phylogenetic network construction, the optimal substitution model for each MSA, and subsequent optimal model parameters were calculated using IQ-TREE version 1.6.3 [43]. Phylogenetic networks for each of the alignments were generated using Splitstree 4 [44] using the optimal substitution model and parameters calculated by IQ-TREE. Maximum likelihood trees were generated using RAxMLGUI (ver. 1.3) using the GTRCATI option with 1000 bootstrap replicates [45].

### Genomic nucleotide distance and clade cutoff calculations

To determine clade cutoff parameters, pairwise distances were first calculated using the genomic MSAs without outgroups. The genomic MSAs without outgroups were used in order to minimize alignment gaps usually created by including an outgroup sequence. A statistical description of establishing clades using genomic nucleotide distance has been previously described [35]. Briefly a variance analysis framework was used, where the *F* statistic.

was calculated for each dataset and plotted as a curve. Maximum composite likelihood (MCL) pairwise distances were calculated with MEGA 7 rather than uncorrected p-distances as have been used previously [34–37]. Species distance cutoffs were established by using the Old World monkey MSA, followed by graphing the frequency of the pairwise MCL distances using the R software package (ver. 3.4.4) [85]. A kernel density plot was also generated in R to assist in determining the clade cutoff value by finding the trough between the low and high MCL distance populations. Intraspecies clade cutoffs were established in a similar manner, using the core herpes B, and herpes papio MSAs (minus outgroup) respectively.

## Data Availability

The NCBI (https://pubmed.ncbi.nlm.nih.gov/) accession numbers used in this study are found in Table 1. The multiple sequence alignments used for this study are available for download at https://brandt.ophth.wisc.edu/data-sets/.

## Abbreviations

BSL-4: Biosafety level 4
CDC: Centers for disease control
Cyno: Cynomologous
FHV-1: *Feline alphaherpesvirus 1*
GMYC: Generalized mixed Yule coalescent
ICNV: International Committee on Nomenclature of Viruses
ICTV: International Committee for Taxonomy of Viruses
isDDH: in silico DNA-DNA hybridization
LD_50_: Lethal dose, 50%
McHV-1: *Macacine alphaherpesvirus 1*; herpes B
MCL: Maximum composite likelihood
ML: Maximum likelihood
MSA: Multiple sequence alignment
NGS: Next-generation sequencing
OWM: Old world monkeys
PaHV-2: *Papiine alphaherpesvirus 2*; herpes papio
PCV2: Porcine circovirus type 2
PFU: Plaque forming unit
SA8: Simian agent 8; *Cercopithecine alphaherpesvirus 2*

## References

[1] Sheppard M, May JT (1989). Location and characterization of the bovine herpesvirus type 2 thymidine kinase gene. J Gen Virol.

[2] Babra B, Watson G, Xu W, Jeffrey BM, Xu JR, Rockey DD, Rohrmann GF, Jin L (2012). Analysis of the genome of leporid herpesvirus 4. Virology.

[3] Sasaki M, Setiyono A, Handharyani E, Kobayashi S, Rahmadani I, Taha S, Adiani S, Subangkit M, Nakamura I, Sawa H (2014). Isolation and characterization of a novel alphaherpesvirus in fruit bats. J Virol.

[4] Vaz PK, Mahony TJ, Hartley CA, Fowler EV, Ficorilli N, Lee SW, Gilkerson JR, Browning GF, Devlin JM (2016). The first genome sequence of a metatherian herpesvirus: Macropodid herpesvirus 1. BMC Genomics.

[5] Mahony TJ, Smith GA, Thomson DM (1999). Macropodid herpesviruses 1 and 2 occupy unexpected molecular phylogenic positions within the Alphaherpesvirinae. J Gen Virol.

[6] Holden FPGaM (1933). The herpes encephalitis problem, II. J Infect Dis.

[7] Holden FPGaM (1933). Isolation of herpes virus from several cases of epidemic encephalitis. Proc Soc Exp Biol Med.

[8] Cohen JI, Davenport DS, Stewart JA, Deitchman S, Hilliard JK, Chapman LE, Group BVW (2002). Recommendations for prevention of and therapy for exposure to B virus (cercopithecine herpesvirus 1). Clin Infect Dis.

[9] B Virus (herpes B, monkey B virus, herpesvirus simiae, and herpesvirus B)**.** https://www.cdc.gov/herpesbvirus/index.html.Accessed 19 Sept 2019.

[10] Hilliard J. Monkey B virus. In: Arvin AC-FG, Mocarski E, et al, editors. Human herpesviruses: Biology, therapy, and immunoprophylaxis. 1st ed. Cambridge: Cambridge University Press; 2007. 21348071

[11] Eberle R, Jones-Engel L (2018). Questioning the extreme neurovirulence of monkey B virus (Macacine alphaherpesvirus 1). Adv Virol.

[12] Eberle R, Maxwell LK, Nicholson S, Black D, Jones-Engel L (2017). Genome sequence variation among isolates of monkey B virus (Macacine alphaherpesvirus 1) from captive macaques. Virology.

[13] Ohsawa K, Black D, Ohsawa M, Eberle R (2014). Genome sequence of a pathogenic isolate of monkey B virus (species Macacine herpesvirus 1). Arch Virol.

[14] Perelygina L, Zhu L, Zurkuhlen H, Mills R, Borodovsky M, Hilliard JK (2003). Complete sequence and comparative analysis of the genome of herpes B virus (Cercopithecine herpesvirus 1) from a rhesus monkey. J Virol.

[15] Li J, Han K, Xing J, Kim HS, Rogers J, Ryder OA, Disotell T, Yue B, Batzer MA (2009). Phylogeny of the macaques (Cercopithecidae: Macaca) based on Alu elements. Gene.

[16] Mayr E (1942). Systematics and the origin of species.

[17] de Queiroz K (2005). Ernst Mayr and the modern concept of species. Proc Natl Acad Sci U S A.

[18] Casto AM, Roychoudhury P, Xie H, Selke S, Perchetti GA, Wofford H, Huang ML, Verjans G, Gottlieb GS, Wald A, et al. Large, stable, contemporary interspecies recombination events in circulating human herpes simplex viruses. J Infect Dis. 2020;221(8):1271–9. 10.1093/infdis/jiz199. 10.1093/infdis/jiz199 PMC7325804 31016321

[19] Burrel S, Boutolleau D, Ryu D, Agut H, Merkel K, Leendertz FH, Calvignac-Spencer S (2017). Ancient recombination events between human herpes simplex viruses. Mol Biol Evol.

[20] Koelle DM, Norberg P, Fitzgibbon MP, Russell RM, Greninger AL, Huang ML, Stensland L, Jing L, Magaret AS, Diem K (2017). Worldwide circulation of HSV-2 x HSV-1 recombinant strains. Sci Rep.

[21] Pringle CR (1965). Evidence of genetic recombination in foot-and-mouth disease virus. Virology.

[22] Ledinko N (1963). Genetic recombination with poliovirus type 1. Studies of crosses between a normal horse serum-resistant mutant and several guanidine-resistant mutants of the same strain. Virology.

[23] Ledinko N (1976). Temperature-sensitive mutants of type 12 adenovirus defective in a late function: protein synthesis and evidence for recombination between mutants in complementation group D. J Gen Virol.

[24] Kolbourne ED (1968). Recombination of influenza A viruses of human and animal origin. Science.

[25] Wildy P (1955). Recombination with herpes simplex virus. J Gen Microbiol.

[26] Lee K, Kolb AW, Sverchkov Y, Cuellar JA, Craven M, Brandt CR (2015). Recombination analysis of herpes simplex virus type 1 reveals a bias towards GC content and the inverted repeat regions. J Virol.

[27] De Queiroz K (2007). Species concepts and species delimitation. Syst Biol.

[28] Winker K (2005). Sibling species were first recognized by William Derham (1718). Auk.

[29] Struck TH, Feder JL, Bendiksby M, Birkeland S, Cerca J, Gusarov VI, Kistenich S, Larsson KH, Liow LH, Nowak MD (2018). Finding evolutionary processes hidden in cryptic species. Trends Ecol Evol.

[30] Schloss PD, Handelsman J (2006). Toward a census of bacteria in soil. PLoS Comput Biol.

[31] Acinas SG, Klepac-Ceraj V, Hunt DE, Pharino C, Ceraj I, Distel DL, Polz MF (2004). Fine-scale phylogenetic architecture of a complex bacterial community. Nature.

[32] Meier-Kolthoff JP, Auch AF, Klenk HP, Goker M (2013). Genome sequence-based species delimitation with confidence intervals and improved distance functions. BMC Bioinformatics.

[33] Tang CQ, Humphreys AM, Fontaneto D, Barraclough TG, Paradis E (2014). Effects of phylogenetic reconstruction method on the robustness of species delimitation using single-locus data. Methods Ecol Evol.

[34] Lewin AC, Kolb AW, McLellan GJ, Bentley E, Bernard KA, Newbury SP, Brandt CR (2018). Genomic, recombinational and phylogenetic characterization of global feline Herpesvirus 1 isolates. Virology.

[35] Kolb AW, Lewin AC, Moeller Trane R, McLellan GJ, Brandt CR (2017). Phylogenetic and recombination analysis of the herpesvirus genus varicellovirus. BMC Genomics.

[36] Segales J, Olvera A, Grau-Roma L, Charreyre C, Nauwynck H, Larsen L, Dupont K, McCullough K, Ellis J, Krakowka S (2008). PCV-2 genotype definition and nomenclature. Vet Rec.

[37] Xiao CT, Halbur PG, Opriessnig T (2015). Global molecular genetic analysis of porcine circovirus type 2 (PCV2) sequences confirms the presence of four main PCV2 genotypes and reveals a rapid increase of PCV2d. J Gen Virol.

[38] Malherbe H, Strickland-Cholmley M (1969). Simian herpesvirus SA8 from a baboon. Lancet.

[39] Malherbe H, Strickland-Cholmley M (1969). Virus from baboons. Lancet.

[40] Tyler SD, Peters GA, Severini A (2005). Complete genome sequence of cercopithecine herpesvirus 2 (SA8) and comparison with other simplexviruses. Virology.

[41] Thompson SA, Hilliard JK, Kittel D, Lipper S, Giddens WE, Black DH, Eberle R (2000). Retrospective analysis of an outbreak of B virus infection in a colony of DeBrazza's monkeys (Cercopithecus neglectus). Comp Med.

[42] Katoh K, Standley DM (2013). MAFFT multiple sequence alignment software version 7: improvements in performance and usability. Mol Biol Evol.

[43] Kalyaanamoorthy S, Minh BQ, Wong TKF, von Haeseler A, Jermiin LS (2017). ModelFinder: fast model selection for accurate phylogenetic estimates. Nat Methods.

[44] Huson DH, Bryant D (2006). Application of phylogenetic networks in evolutionary studies. Mol Biol Evol.

[45] Berger SA, Krompass D, Stamatakis A (2011). Performance, accuracy, and web server for evolutionary placement of short sequence reads under maximum likelihood. Syst Biol.

[46] Kumar S, Stecher G, Tamura K (2016). MEGA7: molecular evolutionary genetics analysis version 7.0 for bigger datasets. Mol Biol Evol.

[47] McGeoch DJ, Dolan A, Ralph AC (2000). Toward a comprehensive phylogeny for mammalian and avian herpesviruses. J Virol.

[48] Pedersen K, Turnage CT, Gaston WD, Arruda P, Alls SA, Gidlewski T (2018). Pseudorabies detected in hunting dogs in Alabama and Arkansas after close contact with feral swine (Sus scrofa). BMC Vet Res.

[49] Fukushi H, Tomita T, Taniguchi A, Ochiai Y, Kirisawa R, Matsumura T, Yanai T, Masegi T, Yamaguchi T, Hirai K (1997). Gazelle herpesvirus 1: a new neurotropic herpesvirus immunologically related to equine herpesvirus 1. Virology.

[50] Loomis MR, O'Neill T, Bush M, Montali RJ (1981). Fatal herpesvirus infection in patas monkeys and a black and white colobus monkey. J Am Vet Med Assoc.

[51] Wilson RB, Holscher MA, Chang T, Hodges JR (1990). Fatal Herpesvirus simiae (B virus) infection in a patas monkey (Erythrocebus patas). J Vet Diagn Investig.

[52] Coulibaly C, Hack R, Seidl J, Chudy M, Itter G, Plesker R (2004). A natural asymptomatic herpes B virus infection in a colony of laboratory brown capuchin monkeys (Cebus apella). Lab Anim.

[53] Sekulin K, Jankova J, Kolodziejek J, Huemer HP, Gruber A, Meyer J, Nowotny N (2010). Natural zoonotic infections of two marmosets and one domestic rabbit with herpes simplex virus type 1 did not reveal a correlation with a certain gG-, gI- or gE genotype. Clin Microbiol Infect.

[54] Simon MA, Daniel MD, Lee-Parritz D, King NW, Ringler DJ (1993). Disseminated B virus infection in a cynomolgus monkey. Lab Anim Sci.

[55] Parrish CR, Holmes EC, Morens DM, Park EC, Burke DS, Calisher CH, Laughlin CA, Saif LJ, Daszak P (2008). Cross-species virus transmission and the emergence of new epidemic diseases. Microbiol Mol Biol Rev.

[56] Jin MJ, Hui H, Robertson DL, Muller MC, Barre-Sinoussi F, Hirsch VM, Allan JS, Shaw GM, Sharp PM, Hahn BH (1994). Mosaic genome structure of simian immunodeficiency virus from west African green monkeys. EMBO J.

[57] Faria NR, Suchard MA, Rambaut A, Streicker DG, Lemey P (2013). Simultaneously reconstructing viral cross-species transmission history and identifying the underlying constraints. Philos Trans R Soc Lond Ser B Biol Sci.

[58] Ram MS, Marne M, Gaur A, Kumara HN, Singh M, Kumar A, Umapathy G (2015). Pre-historic and recent vicariance events shape genetic structure and diversity in endangered lion-tailed macaque in the Western Ghats: implications for conservation. PLoS One.

[59] Erinjery JJ, Kavana TS, Singh M (2017). Behavioural variability in macaques and langurs of the Western Ghats, India. Folia Primatol (Basel).

[60] Matthews REF. The history of virus taxonomy. In: Matthews REF, editor. A critical appraisal of viral taxonomy. Boca Raton: CRC Press; 1983. p. 256.

[61] Lwoff A (1964). The new provisional committee on nomenclature of viruses. Int Bull Bact Nomencl Taxonomy.

[62] Regenmortel MHV. Virus species. In: M.F. Claridge HADaMRW, editor. Species: The units of biodiversity. London: Chapman and Hall; 1997. p. 549.

[63] ICTV Information. https://talk.ictvonline.org/information/w/ictv-information/383/ictv-code. Accessed 12 Oct 2019.

[64] Solis-Lemus C, Knowles LL, Ane C (2015). Bayesian species delimitation combining multiple genes and traits in a unified framework. Evolution.

[65] Aiewsakun P, Adriaenssens EM, Lavigne R, Kropinski AM, Simmonds P (2018). Evaluation of the genomic diversity of viruses infecting bacteria, archaea and eukaryotes using a common bioinformatic platform: steps towards a unified taxonomy. J Gen Virol.

[66] Sukumaran J, Knowles LL (2017). Multispecies coalescent delimits structure, not species. Proc Natl Acad Sci U S A.

[67] Funk WC, Caminer M, Ron SR (2012). High levels of cryptic species diversity uncovered in Amazonian frogs. Proc Biol Sci.

[68] Winterbottom R, Hanner RH, Burridge M, Zur M (2014). A cornucopia of cryptic species - a DNA barcode analysis of the gobiid fish genus Trimma (Percomorpha, Gobiiformes). Zookeys.

[69] Surveswaran S, Gowda V, Sun M (2018). Using an integrated approach to identify cryptic species, divergence patterns and hybrid species in Asian ladies’ tresses orchids (Spiranthes, Orchidaceae). Mol Phylogenet Evol.

[70] Saitoh T, Sugita N, Someya S, Iwami Y, Kobayashi S, Kamigaichi H, Higuchi A, Asai S, Yamamoto Y, Nishiumi I (2015). DNA barcoding reveals 24 distinct lineages as cryptic bird species candidates in and around the Japanese archipelago. Mol Ecol Resour.

[71] Hanelt B, Schmidt-Rhaesa A, Bolek MG (2015). Cryptic species of hairworm parasites revealed by molecular data and crowdsourcing of specimen collections. Mol Phylogenet Evol.

[72] Crespo A, Lumbsch HT (2010). Cryptic species in lichen-forming fungi. IMA Fungus.

[73] Hahn MW, Huymann LR, Koll U, Schmidt J, Lang E, Hoetzinger M (2017). Polynucleobacter wuianus sp. nov., a free-living freshwater bacterium affiliated with the cryptic species complex PnecC. Int J Syst Evol Microbiol.

[74] Palacios G, Savji N, Travassos da Rosa A, Desai A, Sanchez-Seco MP, Guzman H, Lipkin WI, Tesh R (2013). Characterization of the Salehabad virus species complex of the genus Phlebovirus (Bunyaviridae). J Gen Virol.

[75] Gundacker ND, Carrera JP, Castillo M, Diaz Y, Valenzuela J, Tamhane A, Moreno B, Pascale JM, Tesh RB, Lopez-Verges S (2017). Clinical manifestations of Punta Toro virus species complex infections, Panama, 2009. Emerg Infect Dis.

[76] Ottenburghs J (2019). Multispecies hybridization in birds. Avian Res.

[77] Moraes AP, Chinaglia M, Palma-Silva C, Pinheiro F (2013). Interploidy hybridization in sympatric zones: the formation of Epidendrum fulgens x E. puniceoluteum hybrids (Epidendroideae, Orchidaceae). Ecol Evol.

[78] von Holdt BM, Cahill JA, Fan Z, Gronau I, Robinson J, Pollinger JP, Shapiro B, Wall J, Wayne RK (2016). Whole-genome sequence analysis shows that two endemic species of North American wolf are admixtures of the coyote and gray wolf. Sci Adv.

[79] Selz OM, Seehausen O (2019). Interspecific hybridization can generate functional novelty in cichlid fish. Proc Biol Sci.

[80] Dutch RE, Bianchi V, Lehman IR (1995). Herpes simplex virus type 1 DNA replication is specifically required for high-frequency homologous recombination between repeated sequences. J Virol.

[81] Pandey U, Renner DW, Thompson RL, Szpara ML, Sawtell NM (2017). Inferred father-to-son transmission of herpes simplex virus results in near-perfect preservation of viral genome identity and in vivo phenotypes. Sci Rep.

[82] Ruebner BH, Kevereux D, Rorvik M, Espana C, Brown JF (1975). Ultrastructure of Herpesvirus simiae (Herpes B ivurs). Exp Mol Pathol.

[83] Katoh K, Toh H (2010). Parallelization of the MAFFT multiple sequence alignment program. Bioinformatics.

[84] Larkin MA, Blackshields G, Brown NP, Chenna R, McGettigan PA, McWilliam H, Valentin F, Wallace IM, Wilm A, Lopez R (2007). Clustal W and Clustal X version 2.0. Bioinformatics.

[85] Team RC (2013). R: A language and environment for statistical computing. R Foundation for statistical computing.

